# Assessment of endothelial function by brachial artery flow mediated dilatation in microvascular disease

**DOI:** 10.1186/1476-7120-9-40

**Published:** 2011-12-09

**Authors:** Otikunta Adikesava Naidu, Durgaprasad Rajasekhar, SAA Latheef

**Affiliations:** 1 Department of Cardiology, Osmania General Hospital and Osmania Medical College, Hyderabad, Andhra Pradesh, India; 2 Department of Cardiology, Sri Venkateswara Institute of Medical Sciences, Tirupati, Andhra Pradesh, India; 3 Department of Biochemistry and CREBB Centre, School of Life Sciences, University of Hyderabad, Hyderabad, Andhra Pradesh, India

## Abstract

**Background:**

Cardiac syndrome X is an important therapeutic and diagnostic challenge to physician. Study of Csx patients may help to understand the pathophysiology of coronary microcirculation and to gain an insight on the management of these group patients.

**Methods:**

We measured the flow mediated dilation of the brachial artery both endothelium dependent and independent vasodilatation by high resolution ultrasound in 30 cardiac syndrome X patients and matched with 30 healthy control subjects.

**Results:**

Significantly decreased flow mediated dilatation was observed in patients when compared to control (9.42 ± 7.20 vs 21.11 ± 9.16 p < 0.01) but no significant difference was observed between groups in response to nitroglycerin (25.39 ± 6.82 vs 28.87 ± 8.69). Receiver operator characteristic analysis showed that value of < 11.11 had sensitivity of 80%, specificity 86.67%, positive predictive value 76.66%, negative predictive value 83.33%. In total, 46% of subjects had endothelial dysfunction and of them, CSX subjects had higher prevalence (76% vs 16% p < 0.01) than control subjects. Higher mean values of body mass index, systolic blood pressure and diastolic blood pressure was observed in subjects with FMD < 11.11 than > 11.11(p < 0.01). In logistic regression analysis, FMD was significantly associated with systolic blood pressure (Odds ratio 1.122 95% CI 1.053-1.196 p < 0.01) and body mass index (Odds 1.248 95%CI 0.995-1.56 p < 0.05).

**Conclusions:**

The study suggests impairment of endothelial function in cardiac syndrome X patients. Increased Systolic blood pressure and body mass index may increase the risk of impairment of endothelial function in this group of patients.

## Introduction

Cardiac syndrome X (Csx) is defined by the presence of angina-like chest pain, a positive response to stress testing and angiographically normal coronary arteries [1] Csx represent a heterogeneous entity in terms of clinically and pathophysiologically [2], interesting because around 20% of the patients with angina chest pain undergoing coronary angiography have normal coronary arteries [3] and pose an important diagnostic and therapeutic challenges to the physicians. Study of CSX patients may help to understand the pathophysiology of coronary microcirculation and cardiac pain [4] Microvascular dysfunction has been observed in CSX patients [5,6]

In the absence of established mechanism, multiple pathogenic mechanisms have been proposed for Csx includes: abnormal coronary flow reserve, insulin resistance, abnormal autonomic control, enhanced sodium-hydrogen exchange activity, abnormal cardiac sensitivity, microvascular spasm, extracardiac causes, psychological abnormalities, myocardial ischemia and abnormal pain perception [2,7]. Evaluation of endothelial function has prognostic value [8] Endothelial function assessment is useful in identifying early atherosclerosis changes and the detection of the severity as well [9]. Given the central role of the endothelium in the development and clinical course of atherosclerosis, endothelial function testing may serve as a useful biomarker of atherosclerosis [10]. Interventions that improve endothelial function also decrease cardiovascular events [11]. Vessel function may play a very important role in determining cardiovascular risk, over and above the risk conveyed by a structural impediment to flow such as a large plaque [12] In this study, we have made an attempt to evaluate the endothelial function in brachial artery of CSX patients using high resolution B-mode ultrasound and to correlate with coronary risk factors.

## Materials and methods

In this study, 30 patients with history of angina like chest pain, reported positive on tread mill test and with normal coronaries on coronary angiography, were diagnosed as Csx patients. Thirty age matched subjects, who were diagnosed healthy based on ECG and echocardiogram were treated as control. Informed consent was obtained from each of the patients and health controls following ethical guidelines of the 1975 Declaration of Helsinki. This study was approved by Institutional Ethics Committee of Sri Venkateswara Institute of Medical Sciences, Tirupti, Andhra Pradesh, India. The age of both groups of subjects ranged from 40-60 years. There were 20 male and 10 female in CSx; and 17 male and 13 female subjects in control group. Details of age, history of hypertension, ischemic heart disease, type 2 diabetes and smoking were enquired. Subjects were examined clinically for pulse, blood pressure, respiratory, cardiovascular and central nervous system followed by ECG and Echocardiogram evaluation. Height and weight were measured. Body mass index was calculated.

Blood samples were drawn after 12 hr fasting. The samples were assayed for glucose, total cholesterol, triglyceride, HDL cholesterol, blood urea and creatinine. LDL cholesterol was calculated as per Fridewald et al [13]. Endothelial function of subjects was evaluated by Doppler study on right brachial artery following Celermajer et al [14]. The subjects were requested to abstain from alcohol, caffeine and smoking for 8 hours before attending the procedure. The procedure was performed by 7.5 MHZ linear array tranducer with image point HX using Ultra sound equipment of Agilent technology, India. Scans of subjects were obtained at rest, during reactive hyperemia and again at rest.

The subjects were asked to lie quietly for at least 10 min before the first scan. The brachial artery was scanned in longitudinal section 2 cm above the elbow, and the center of the artery was identified when the clearest picture of the anterior and posterior intimal layers were obtained. The transmit zone was set to the depth of the near wall, because of the greater difficulty in evaluating the "M" line (the interface between the media and adventia) of the near wall as compared with that of the far wall. Depth and gain settings were set to optimize images of interface between the lumen and the arterial wall, and the obtained images were magnified. Settings for operating the machine were not changed during the study.

When a satisfactory transducer position was found, the skin was marked and the arm was kept in the same position throughout the study. A resting scan was obtained. The arterial diameter was measured. Increased flow was induced by the inflation of a sphygmomanometer cuff placed around the forearm (distal to the scanned part of the artery) at a pressure of 200 mmHg for 4.5 min, followed by release. A second scan was performed 90 s after deflation of the cuff. The diameter of the artery was measured at the peak of R wave (corresponding to end diastole). Flow-mediated dilation was calculated. Average of three observations was recorded. Flow-mediated dilatation was presented as the percent change from baseline to hyperemia. Fifteen minutes were allowed for vessel recovery and then a further resting scan was taken. After that sublingual glyceryl trinitrate (GTN-200 mics/puff) was administered and ten minutes later, the last scan was done. ECG was monitored through the scans and the artery diameter was measured at the peak of R wave (corresponding to end diastole). An average of 3 values was taken for each measurement. FMD (flow mediated vasodilatation) and NMD (nitrate mediated vasodilatation) in syndrome X was compared with the data of healthy control subjects. FMD was calculated using the formula FMD = (d2-d1) × 100/d1. Where d1 is the brachial artery diameter at baseline, d2 is brachial artery diameter after 90 seconds of cuff release. Changes in the diameter after both interventions i.e. after stress and GTN, was expressed as percentage change from the pre-treatment value.

### Statistical analysis

Means were compared with student 't" test and categorical variables with chi-square. Logistic regression was done to find the relation between dependent and independent variables. FMD Cutt-off values were determined using receiver operator curve analysis.

## Results

Higher mean systolic, diastolic blood pressure and lower glucose and % FMD were observed in Csx patients when compared to the control subjects (p < 0.01). Lower mean % of FMD was observed in male than female subjects (12.35 ± 7.73 vs. 19.96 ± 11.71p < 0.01). Lower mean % of FMD with increasing BMI was observed in both cases and control (in cases; BMI < 25 13.68 ± 6.58 vs. 6.58 p < 0.00; control: 23.68 ± 9.09 vs 15. 97 ± 7.19 p < 0.00) subjects (Table 1).

**Table 1:** Clinical characteristics of the subjects

Variables	CSX(n = 30)	Control(n = 30)
**Age (years)**	48.76 ± 6.57	47.70 ± 6.79

**Body mass index**	27.52 ± 5.79	24.35 ± 3.09*

**Systolic blood pressure (mmHg)**	144.73 ± 12.10	114.00 ± 8.94*

**Diastolic blood pressure (mmHg)**	93.80 ± 9.88	74.86 ± 5.39*

**Glucose (mg/dl)**	75.70 ± 21.31	88.40 ± 21.38*

**Total Cholesterol (mg/dl)**	179.00 ± 15.52	174.73 ± 20.58

**Triglycerides (mg/dl)**	131.66 ± 39.80	138.16 ± 40.86

**HDL cholesterol (mg/dl)**	43.70 ± 8.26	42.26 ± 7.01

**LDL cholesterol (mg/dl)**	113.16 ± 18.51	107 ± 21.12

**VLDL cholesterol (mg/dl)**	26.26 ± 7.82	25.56 ± 7.53

**Serum Creatinine (mg/dl)**	0.88 ± 0.20	0.84 ± 0.17

**Blood urea(mg/dl)**	22.80 ± 7.43	22.50 ± 6.31

**Mean % of FMD**	9.42 ± 7.20	21.11 ± 9.16*

**Mean % NMD**	25.39 ± 6.82	28.87 ± 8.69

*p < 0.01

Higher mean body mass index, systolic blood pressure and diastolic blood pressure was observed in FMD < 11.11 when compared to FMD > 11.11(p < 0.01) (table 2).

**Table 2:** Clinical characteristics of the subjects with respect to FMD

Variables	FMD < 11.11(n = 28)	FMD > 11.11(n = 32)
**Age (years)**	49.21 ± 6.18	47.37 ± 7.01

**Body mass index**	28.44 ± 5.51	23.74 ± 2.84*

**Systolic blood pressure (mmHg)**	143.28 ± 15.30	117.18 ± 11.70*

**Diastolic blood pressure (mmHg)**	92.92 ± 10.46	76.81 ± 8.46*

**Glucose (mg/dl)**	77.78 ± 21.31	85.78 ± 22.45

**Total Cholesterol (mg/dl)**	176.96 ± 15.27	176.78 ± 20.67

**Triglycerides (mg/dl)**	134.21 ± 36.79	135.53 ± 43.41

**HDL cholesterol (mg/dl)**	42.60 ± 8.10	43.31 ± 7.31

**LDL cholesterol (mg/dl)**	113.00 ± 18.71	107.56 ± 20.91

**VLDL cholesterol (mg/dl)**	26.10 ± 7.10	25.75 ± 8.16

**Serum Creatinine (mg/dl)**	0.89 ± 0.20	0.84 ± 0.17

**Blood urea(mg/dl)**	22.21 ± 7.30	23.03 ± 6.50

*p < 0.01

**Figure 1:**
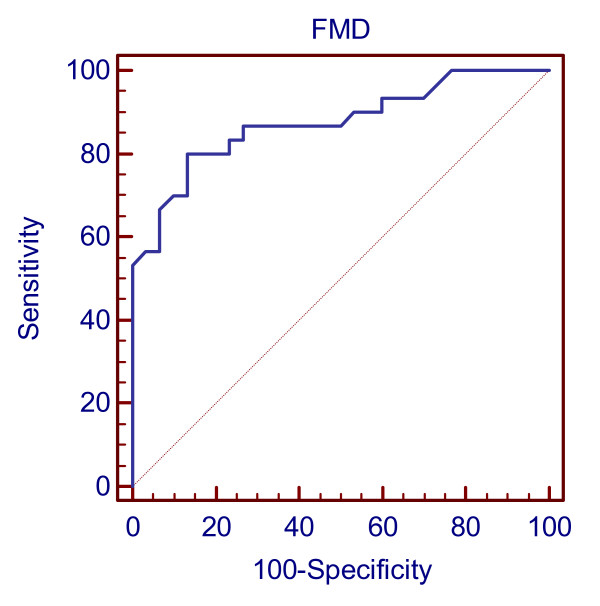
**Receiver operator characteristic (*ROC*) curve for FMD values**. Area under the curve (AUC) = 0.874 95% CI 0.763-0.946 (p < 0.0001)

To determine the sensitivity and specificity of FMD cut-off value in this population, we have performed receiver operator characteristic analysis (ROC). In ROC analysis (Figure 1), we have found that FMD < 11.1 had sensitivity, specificity, positive predictive value and negative predictive values in the following percentage 80%, 86.67%, 76.66% and 83.33% respectively (Figure 1).

In subjects with CSX, 77% had endothelial dysfunction as indicated by FMD < 11.1 as against 17% in control group (p < 0.01). When assessed in logistic regression analysis, FMD was significantly associated with Csx (Odds ratio 16.42 95% CI 4.569-59.07 p < 0.001).

A logistic regression analysis was done to find out the factors contributing to the microvascular dysfunction in Csx and found that systolic blood pressure (Odds ratio 1.122 95% CI 1.053-1.196 p < 0.01) and body mass index (Odds 1.248 95%CI 0.995-1.56 p < 0.05) were significantly associated.

## Discussion

Endothelium is the monolayer of endothelilal cells lining the lumen of blood vessels [10]. It maintains a balance between vasoactive and vasodilator susbstances and the loss of this function leads to endothelial dysfunction. Insight into the function of endothelium has led to the development of tests for its assessment. All of these tests are based on the vasomotor response of endothelium to vasoactive stimuli. Intracoronary doppler techniques have disadvantages like invasive nature, expensive, relatively inaccessibility, risks inherent with coronary artery, difficulty in extending to larger studies and understanding microvascular pathophysiology and failure to represent true changes in conduit arteries predisposed to atherosclerosis [15,16]. The drawbacks associated with plethysmography are reproducibility, invasive nature, risk of median nerve injury, infection and vascular injury [17]. Vascular tonometry and measurement of vascular stiffness are limited by influence of structural aspects of the vasculature beyond the endothelium.

Non-invasive ultrasound test is based on the measurement of changes in brachial artery diameter in response to reactive hyperemia and is considered as a gold standard for clinical studies on conduit artery endothelial biology [18]. It is repeatable and reproducible, reflects important biology, has some data to support its predictability and is useful in serial studies of disease reversibility [12]. Brachial artery endothelium-dependent dilatation was found to be correlated with coronary circulation in the same patient [12,19]. A single measurement of endothelial function in both the coronary and peripheral circulation can be of prognostic value in both normal and coronary heart disease patients [16,20,21].

Several studies have observed lower flow mediated dilation in CSX patients when compared to healthy controls [22-26] but few studies [24-26] observed no difference in endothelium independent vasodilatation. In the present study also, we have observed lower endothelium dependent flow mediated dilation in CSX patients when compared to the healthy control (p < 0.01). In case of glyceryl nitrite dependent dilation, no such difference was observed in our observation. It appears that endothelium dependent vascular dysfunction in CSX group of patients, is probably a generalized phenomenon as observed earlier [27].

Elevated levels of total cholesterol, LDL cholesterol, apolipoprotein B100, Lp(a), aortic and fractional pulse, platelet volume, nocturnal melatonin levels, CRP, reactive oxygen species and hydroperoxides and decreased oestrogen were observed in CSX patients [2,24,28-32]. In the present study, higher mean levels of body mass index, systolic and diastolic blood pressure was observed in CSX patients when compared to the control group.

An area under curve for cut-off value of 11.1 showed a value of 0.874 indicating that ultrasound is useful tool for identifying endothelial impairment in Csx patients and able to predict the impairment in 86% of subjects as shown by specificity of the cut-off value. This cut-off value is observed in a cross-sectional study and has to be established in longitudinal study involving larger sample size. To the best of our knowledge, no study in India reported cutt-off value for flow mediated dilation in Csx patients and this study attempts to propose the cut-off value for flow-mediated dilatation in Indian population. We have assessed the endothelial function manually using hand held probe and this is the limitation of the study.

In the present study, higher mean body mass index, systolic and diastolic blood pressure in FMD < 11.11 than FMD > 11.11 group (p < 0.01) was observed. Systolic blood pressure and body mass index significantly associated with impairment of endothelial function. A 5.77 fold increase in the incidence of hypertension in the lowest than highest FMD quartile was observed over a follow up of 3.5 years in risk factor free post menopausal women [33]. A specific endothelial NO abnormality in microcirculation and conductance vessels of hypertensive subjects has been confirmed in multiple well controlled clinical investiagations [34]. Increase in systemic blood pressure in normotenisve subjects after intravenous infusion of nitric oxide synthase antagonists was observed [35]. Higher blood pressure in endothelial nitric oxide synthase knock out when compared to normal mice, more nitric oxide synthase 3 gene mutations in hypertnsive subjects also established impaired endothelial function [34].

Lower FMD was observed with increase in BMI [36,37]. Nuclear Factor κB Activation contributes to vascular endothelial dysfunction via oxidative stress in overweight/obese middle-aged and older humans [38]. Modest weight loss can improve endothelial function and affect the entire cluster of coronary heart disease risk factors simultaneously [39].

The results of the present study showed higher prevalence of microvascular dysfunction (78%) in the CSx patients when compared to the control subjects and suggest that early detection and management of systolic blood pressure and encouraging weight loss may be helpful in the management of CSX.

## Competing interests

The authors declare that they have no competing interests.

## Authors' contributions

DR designed and interpreted the study; AKN acquired and interpreted the data; SAAL statistical analysis, interpretation and drafted the manuscript. All authors read and approved the final manuscript.
